# Supplementary material for: Empirical Analysis of Preferences of Older Adults for Care Facilities in Japan: Focusing on Household Structure and Economic Status
Source: Healthcare (Basel). 2023 Jun 25;11(13):1843. doi: 10.3390/healthcare11131843 (PMC10340401; doi:10.3390/healthcare11131843)
Supplement: Supplementary file 1 [file healthcare-11-01843-s001.zip › healthcare-2432761-supplementary.pdf]

## Supplementary Materials

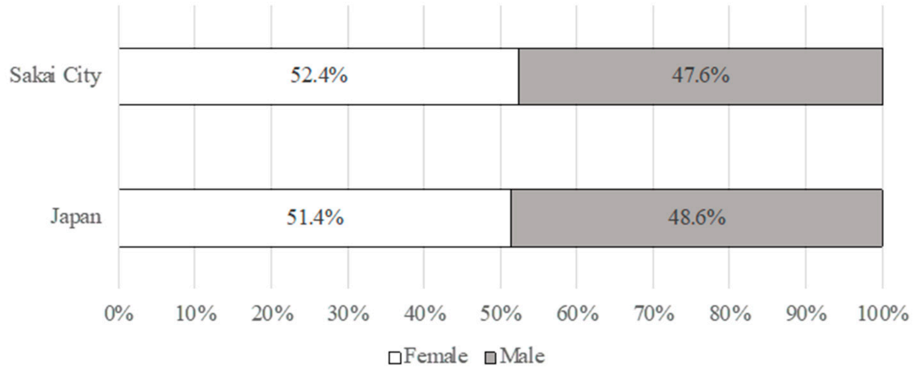


**Figure S1.** Comparison of population structure by sex(2021). Data source: Ministry of Internal Affairs and Communications, Population Estimates (2021) [1]; Sakai City, Population Estimates (2021) [41].

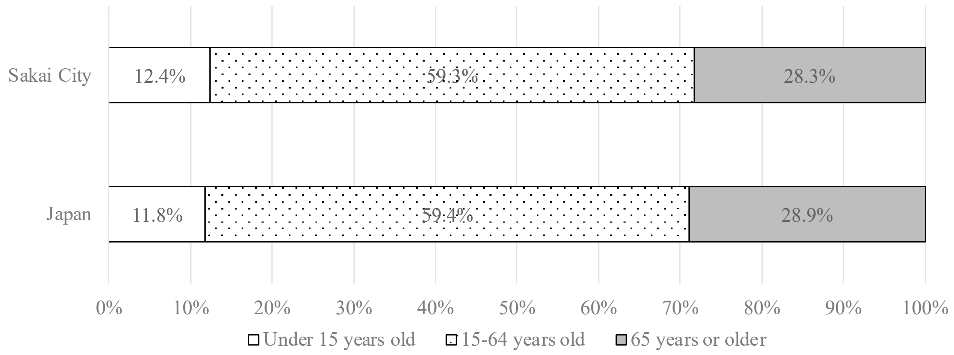


**Figure S2.** Comparison of population structure by age (2021). Data source: Ministry of Internal Affairs and Communications, Population Estimates (2021) [1]; Sakai City, population by age in all cities/ areas (2021) [42].
